# Supplementary material for: Rarity is a more reliable indicator of land-use impacts on soil invertebrate communities than other diversity metrics
Source: eLife. 2020 May 19;9:e52787. doi: 10.7554/eLife.52787 (PMC7237214; doi:10.7554/eLife.52787)
Supplement: Figure 1—source data 1. [file elife-52787-fig1-data1.docx]

Figure 1-source data 1

A. Results of PERMANOVA tests for community composition differences between land-use categories, for overall soil invertebrates and for the main phyla detected, based on Jaccard distances.

| **Taxonomic group** | **Term** | **Df** | **SumSq** | **MeanSq** | **F value** | **R^2^** | **Pr(>F)** |
| --- | --- | --- | --- | --- | --- | --- | --- |
| Overall | Land use | 4 | 3.1429 | 0.7857 | 1.8047 | 0.1058 | ≤ 0.001 |
|  | Residuals | 61 | 26.5577 | 0.4354 |  | 0.8942 |  |
|  | Total | 65 | 29.7007 |  |  | 1 |  |
| Annelida | Land use | 4 | 2.6278 | 0.6570 | 1.4465 | 0.1139 | ≤ 0.001 |
|  | Residuals | 44 | 20.4384 | 0.4542 |  | 0.8861 |  |
|  | Total | 48 | 23.0662 |  |  | 1 |  |
| Arthropoda | Land use | 4 | 2.9158 | 0.7290 | 1.6534 | 0.0978 | ≤ 0.001 |
|  | Residuals | 61 | 26.8930 | 0.4409 |  | 0.9022 |  |
|  | Total | 65 | 29.8088 |  |  | 1 |  |
| Mollusca | Land use | 4 | 3.3019 | 0.8255 | 1.9735 | 0.1521 | ≤ 0.001 |
|  | Residuals | 40 | 18.4040 | 0.4183 |  | 0.8479 |  |
|  | Total | 44 | 21.7059 |  |  | 1 |  |
| Nematoda | Land use | 4 | 3.6517 | 0.9129 | 2.2877 | 0.1343 | ≤ 0.001 |
|  | Residuals | 57 | 23.5442 | 0.3991 |  | 0.8657 |  |
|  | Total | 61 | 27.1959 |  |  | 1 |  |
| Rotifera | Land use | 4 | 3.1910 | 0.7978 | 1.7851 | 0.1131 | ≤ 0.001 |
|  | Residuals | 56 | 25.0259 | 0.4469 |  | 0.8869 |  |
|  | Total | 60 | 28.2169 |  |  | 1 |  |

B. Results of ANOVA tests for differences in multivariate homogeneity of sample dispersions, mean pairwise beta diversity, and mean pairwise phylogenetic beta diversity (UniFrac distances) between land-use categories, for overall soil invertebrates and for the main phyla detected.

| **Test** | **Taxonomic group** | **Term** | **Df** | **Sum Sq** | **Mean Sq** | **F value** | **Pr(>F)** |
| --- | --- | --- | --- | --- | --- | --- | --- |
| Multivariate homogeneity of sample dispersions | Overall | Groups | 4 | 0.0067 | 0.0017 | 3.59 | 0.011 |
|  |  | Residuals | 61 | 0.0283 | 0.0005 |  |  |
|  | Annelida | Groups | 4 | 0.0128 | 0.0032 | 2.47 | 0.058 |
|  |  | Residuals | 45 | 0.0584 | 0.0013 |  |  |
|  | Arthropoda | Groups | 4 | 0.0068 | 0.0017 | 4.51 | 0.003 |
|  |  | Residuals | 61 | 0.0231 | 0.0004 |  |  |
|  | Mollusca | Groups | 4 | 0.0367 | 0.0092 | 2.73 | 0.041 |
|  |  | Residuals | 44 | 0.1482 | 0.0034 |  |  |
|  | Nematoda | Groups | 4 | 0.0106 | 0.0027 | 1.48 | 0.221 |
|  |  | Residuals | 59 | 0.1062 | 0.0018 |  |  |
|  | Rotifera | Groups | 4 | 0.0038 | 0.0010 | 1.20 | 0.321 |
|  |  | Residuals | 56 | 0.0448 | 0.0008 |  |  |
| Beta diversity | Overall | Land use | 4 | 0.0607 | 0.0152 | 14.99 | < 0.001 |
|  |  | Residuals | 442 | 0.4475 | 0.0010 |  |  |
|  | Annelida | Land use | 4 | 0.1837 | 0.0459 | 13.14 | < 0.001 |
|  |  | Residuals | 441 | 1.5414 | 0.0035 |  |  |
|  | Arthropoda | Land use | 4 | 0.0558 | 0.0140 | 15.84 | < 0.001 |
|  |  | Residuals | 442 | 0.3893 | 0.0009 |  |  |
|  | Mollusca | Land use | 4 | 0.5035 | 0.1259 | 21.12 | < 0.001 |
|  |  | Residuals | 441 | 2.6280 | 0.0060 |  |  |
|  | Nematoda | Land use | 4 | 0.2008 | 0.0502 | 13.26 | < 0.001 |
|  |  | Residuals | 442 | 1.6738 | 0.0038 |  |  |
|  | Rotifera | Land use | 4 | 0.0038 | 0.0009 | 0.46 | 0.765 |
|  |  | Residuals | 442 | 0.9080 | 0.0021 |  |  |
| Phylogenetic beta diversity | Overall | Land use | 4 | 0.0599 | 0.0150 | 8.68 | < 0.001 |
|  |  | Residuals | 442 | 0.7633 | 0.0017 |  |  |
|  | Annelida | Land use | 4 | 0.6199 | 0.1550 | 13.43 | < 0.001 |
|  |  | Residuals | 441 | 5.0882 | 0.0115 |  |  |
|  | Arthropoda | Land use | 4 | 0.0436 | 0.0109 | 5.72 | < 0.001 |
|  |  | Residuals | 442 | 0.8426 | 0.0019 |  |  |
|  | Mollusca | Land use | 4 | 0.6497 | 0.1624 | 15.13 | < 0.001 |
|  |  | Residuals | 441 | 4.7343 | 0.0107 |  |  |
|  | Nematoda | Land use | 4 | 0.2509 | 0.0627 | 10.86 | < 0.001 |
|  |  | Residuals | 442 | 2.5513 | 0.0058 |  |  |
|  | Rotifera | Land use | 4 | 0.0478 | 0.0120 | 3.04 | 0.017 |
|  |  | Residuals | 442 | 1.7380 | 0.0039 |  |  |
