# Supplementary material for: Rarity is a more reliable indicator of land-use impacts on soil invertebrate communities than other diversity metrics
Source: eLife. 2020 May 19;9:e52787. doi: 10.7554/eLife.52787 (PMC7237214; doi:10.7554/eLife.52787)
Supplement: Figure 3—source data 1. [file elife-52787-fig3-data1.docx]

Figure 3-source data 1

A. Results of ANOVA tests for differences in overall soil invertebrate biodiversity between land-use categories. Tests resulting in *P*-values ≤ 0.05 are highlighted.

| **Metric** | **Term** | **Df** | **Sum Sq.** | **Mean Sq.** | ***F* stat.** | **R^2^** | ***P*** |
| --- | --- | --- | --- | --- | --- | --- | --- |
| Richness | Land use | 4 | 55916.89 | 13979.22 | 3.56 | 0.18 | 0.011 |
|  | Residuals | 64 | 251189.74 | 3924.84 |  | 0.82 |  |
| Effective Species | Land use | 4 | 2197.93 | 549.48 | 2.24 | 0.12 | 0.074 |
|  | Residuals | 64 | 15694.13 | 245.22 |  | 0.88 |  |
| Rarity | Land use | 4 | 28396.44 | 7099.11 | 8.83 | 0.36 | < 0.001 |
|  | Residuals | 64 | 51436.34 | 803.69 |  | 0.64 |  |
| Phylogenetic Diversity | Land use | 4 | 1674.83 | 418.71 | 3.85 | 0.19 | 0.007 |
|  | Residuals | 64 | 6964.40 | 108.82 |  | 0.81 |  |
| Phylogenetic Rarity | Land use | 4 | 336.43 | 84.11 | 8.51 | 0.35 | < 0.001 |
|  | Residuals | 64 | 632.44 | 9.88 |  | 0.65 |  |
| Mean Pairwise Distance | Land use | 4 | 0.01 | 0.00 | 1.17 | 0.07 | 0.331 |
|  | Residuals | 64 | 0.11 | 0.00 |  | 0.93 |  |

B. Results of ANOVA tests for biodiversity differences between land-use categories for the different invertebrate groups. ‘Non-mites’ consists of Araneae, Opiliones, and Pseudoscorpiones. ‘Other insects’ consists of all insect orders other than Coleoptera, Diptera, Hemiptera, Hymenoptera, and Lepidoptera. Tests resulting in *P*-values ≤ 0.05 are highlighted.

| **Metric** | **Taxon** | **Term** | **Df** | **Sum Sq.** | **Mean Sq.** | ***F* stat.** | **R^2^** | ***P*** |
| --- | --- | --- | --- | --- | --- | --- | --- | --- |
| Richness | Collembola | Land use | 4 | 68.428 | 17.107 | 1.582 | 0.093 | 0.190 |
|  |  | Residuals | 62 | 670.617 | 10.816 |  | 0.907 |  |
|  | Coleoptera | Land use | 4 | 375.040 | 93.760 | 2.702 | 0.146 | 0.038 |
|  |  | Residuals | 63 | 2186.019 | 34.699 |  | 0.854 |  |
|  | Diptera | Land use | 4 | 673.133 | 168.283 | 4.820 | 0.234 | 0.002 |
|  |  | Residuals | 63 | 2199.632 | 34.915 |  | 0.766 |  |
|  | Hymenoptera | Land use | 4 | 374.664 | 93.666 | 6.319 | 0.293 | < 0.001 |
|  |  | Residuals | 61 | 904.199 | 14.823 |  | 0.707 |  |
|  | Lepidoptera | Land use | 4 | 592.900 | 148.225 | 6.176 | 0.282 | < 0.001 |
|  |  | Residuals | 63 | 1511.968 | 23.999 |  | 0.718 |  |
|  | Hemiptera | Land use | 4 | 88.089 | 22.022 | 2.555 | 0.143 | 0.048 |
|  |  | Residuals | 61 | 525.865 | 8.621 |  | 0.857 |  |
|  | other insects | Land use | 4 | 90.397 | 22.599 | 2.671 | 0.149 | 0.040 |
|  |  | Residuals | 61 | 516.043 | 8.460 |  | 0.851 |  |
|  | non-mites | Land use | 4 | 59.827 | 14.957 | 0.932 | 0.057 | 0.451 |
|  |  | Residuals | 62 | 995.039 | 16.049 |  | 0.943 |  |
|  | Mites | Land use | 4 | 181.264 | 45.316 | 1.310 | 0.077 | 0.276 |
|  |  | Residuals | 63 | 2178.736 | 34.583 |  | 0.923 |  |
|  | Malacostraca | Land use | 4 | 1.350 | 0.337 | 0.333 | 0.041 | 0.853 |
|  |  | Residuals | 31 | 31.400 | 1.013 |  | 0.959 |  |
|  | myriapods | Land use | 4 | 5.981 | 1.495 | 0.523 | 0.067 | 0.720 |
|  |  | Residuals | 29 | 82.989 | 2.862 |  | 0.933 |  |
|  | Annelida | Land use | 4 | 88.496 | 22.124 | 1.420 | 0.085 | 0.238 |
|  |  | Residuals | 61 | 950.534 | 15.583 |  | 0.915 |  |
|  | Mollusca | Land use | 4 | 145.167 | 36.292 | 2.362 | 0.134 | 0.063 |
|  |  | Residuals | 61 | 937.317 | 15.366 |  | 0.866 |  |
|  | Nematoda | Land use | 4 | 2023.103 | 505.776 | 2.857 | 0.151 | 0.030 |
|  |  | Residuals | 64 | 11331.187 | 177.050 |  | 0.849 |  |
|  | Platyhelminthes | Land use | 4 | 10.397 | 2.599 | 3.542 | 0.235 | 0.013 |
|  |  | Residuals | 46 | 33.760 | 0.734 |  | 0.765 |  |
|  | Rotifera | Land use | 4 | 1059.272 | 264.818 | 0.830 | 0.050 | 0.511 |
|  |  | Residuals | 63 | 20096.419 | 318.991 |  | 0.950 |  |
|  | Tardigrada | Land use | 4 | 0.286 | 0.071 | 0.172 | 0.025 | 0.951 |
|  |  | Residuals | 27 | 11.214 | 0.415 |  | 0.975 |  |
| Effective Species | Collembola | Land use | 4 | 8.473 | 2.118 | 1.111 | 0.067 | 0.360 |
|  |  | Residuals | 62 | 118.257 | 1.907 |  | 0.933 |  |
|  | Coleoptera | Land use | 4 | 24.017 | 6.004 | 1.707 | 0.098 | 0.160 |
|  |  | Residuals | 63 | 221.645 | 3.518 |  | 0.902 |  |
|  | Diptera | Land use | 4 | 67.922 | 16.981 | 4.365 | 0.217 | 0.004 |
|  |  | Residuals | 63 | 245.091 | 3.890 |  | 0.783 |  |
|  | Hymenoptera | Land use | 4 | 53.899 | 13.475 | 3.036 | 0.166 | 0.024 |
|  |  | Residuals | 61 | 270.695 | 4.438 |  | 0.834 |  |
|  | Lepidoptera | Land use | 4 | 83.670 | 20.917 | 4.206 | 0.211 | 0.004 |
|  |  | Residuals | 63 | 313.306 | 4.973 |  | 0.789 |  |
|  | Hemiptera | Land use | 4 | 3.562 | 0.891 | 0.365 | 0.023 | 0.833 |
|  |  | Residuals | 61 | 148.879 | 2.441 |  | 0.977 |  |
|  | other insects | Land use | 4 | 3.796 | 0.949 | 0.666 | 0.042 | 0.618 |
|  |  | Residuals | 61 | 86.853 | 1.424 |  | 0.958 |  |
|  | non-mites | Land use | 4 | 17.707 | 4.427 | 1.361 | 0.081 | 0.258 |
|  |  | Residuals | 62 | 201.637 | 3.252 |  | 0.919 |  |
|  | mites | Land use | 4 | 40.681 | 10.170 | 2.725 | 0.148 | 0.037 |
|  |  | Residuals | 63 | 235.099 | 3.732 |  | 0.852 |  |
|  | Malacostraca | Land use | 4 | 1.063 | 0.266 | 1.446 | 0.157 | 0.243 |
|  |  | Residuals | 31 | 5.698 | 0.184 |  | 0.843 |  |
|  | myriapods | Land use | 4 | 1.918 | 0.479 | 0.433 | 0.056 | 0.784 |
|  |  | Residuals | 29 | 32.133 | 1.108 |  | 0.944 |  |
|  | Annelida | Land use | 4 | 18.827 | 4.707 | 2.927 | 0.161 | 0.028 |
|  |  | Residuals | 61 | 98.104 | 1.608 |  | 0.839 |  |
|  | Mollusca | Land use | 4 | 34.335 | 8.584 | 2.169 | 0.125 | 0.083 |
|  |  | Residuals | 61 | 241.439 | 3.958 |  | 0.875 |  |
|  | Nematoda | Land use | 4 | 175.160 | 43.790 | 1.559 | 0.089 | 0.196 |
|  |  | Residuals | 64 | 1797.223 | 28.082 |  | 0.911 |  |
|  | Platyhelminthes | Land use | 4 | 2.437 | 0.609 | 2.485 | 0.178 | 0.057 |
|  |  | Residuals | 46 | 11.279 | 0.245 |  | 0.822 |  |
|  | Rotifera | Land use | 4 | 159.456 | 39.864 | 0.762 | 0.046 | 0.554 |
|  |  | Residuals | 63 | 3293.705 | 52.281 |  | 0.954 |  |
|  | Tardigrada | Land use | 4 | 0.952 | 0.238 | 0.855 | 0.112 | 0.503 |
|  |  | Residuals | 27 | 7.513 | 0.278 |  | 0.888 |  |
| Rarity | Collembola | Land use | 4 | 7.128 | 1.782 | 0.807 | 0.049 | 0.526 |
|  |  | Residuals | 62 | 136.937 | 2.209 |  | 0.951 |  |
|  | Coleoptera | Land use | 4 | 282.076 | 70.519 | 6.781 | 0.301 | < 0.001 |
|  |  | Residuals | 63 | 655.159 | 10.399 |  | 0.699 |  |
|  | Diptera | Land use | 4 | 439.481 | 109.870 | 13.263 | 0.457 | < 0.001 |
|  |  | Residuals | 63 | 521.908 | 8.284 |  | 0.543 |  |
|  | Hymenoptera | Land use | 4 | 133.802 | 33.450 | 6.688 | 0.305 | < 0.001 |
|  |  | Residuals | 61 | 305.096 | 5.002 |  | 0.695 |  |
|  | Lepidoptera | Land use | 4 | 261.129 | 65.282 | 8.873 | 0.360 | < 0.001 |
|  |  | Residuals | 63 | 463.539 | 7.358 |  | 0.640 |  |
|  | Hemiptera | Land use | 4 | 29.977 | 7.494 | 3.276 | 0.177 | 0.017 |
|  |  | Residuals | 61 | 139.534 | 2.287 |  | 0.823 |  |
|  | other insects | Land use | 4 | 42.320 | 10.580 | 3.603 | 0.191 | 0.011 |
|  |  | Residuals | 61 | 179.139 | 2.937 |  | 0.809 |  |
|  | non-mites | Land use | 4 | 81.931 | 20.483 | 4.324 | 0.218 | 0.004 |
|  |  | Residuals | 62 | 293.703 | 4.737 |  | 0.782 |  |
|  | mites | Land use | 4 | 44.981 | 11.245 | 1.634 | 0.094 | 0.177 |
|  |  | Residuals | 63 | 433.611 | 6.883 |  | 0.906 |  |
|  | Malacostraca | Land use | 4 | 1.034 | 0.259 | 0.476 | 0.058 | 0.753 |
|  |  | Residuals | 31 | 16.836 | 0.543 |  | 0.942 |  |
|  | myriapods | Land use | 4 | 6.675 | 1.669 | 1.270 | 0.149 | 0.304 |
|  |  | Residuals | 29 | 38.098 | 1.314 |  | 0.851 |  |
|  | Annelida | Land use | 4 | 54.989 | 13.747 | 4.849 | 0.241 | 0.002 |
|  |  | Residuals | 61 | 172.949 | 2.835 |  | 0.759 |  |
|  | Mollusca | Land use | 4 | 40.162 | 10.040 | 2.059 | 0.119 | 0.097 |
|  |  | Residuals | 61 | 297.454 | 4.876 |  | 0.881 |  |
|  | Nematoda | Land use | 4 | 367.625 | 91.906 | 2.891 | 0.153 | 0.029 |
|  |  | Residuals | 64 | 2034.730 | 31.793 |  | 0.847 |  |
|  | Platyhelminthes | Land use | 4 | 3.583 | 0.896 | 2.598 | 0.184 | 0.048 |
|  |  | Residuals | 46 | 15.861 | 0.345 |  | 0.816 |  |
|  | Rotifera | Land use | 4 | 234.352 | 58.588 | 0.735 | 0.045 | 0.571 |
|  |  | Residuals | 63 | 5019.957 | 79.682 |  | 0.955 |  |
|  | Tardigrada | Land use | 4 | 0.617 | 0.154 | 0.571 | 0.078 | 0.686 |
|  |  | Residuals | 27 | 7.300 | 0.270 |  | 0.922 |  |
| Phylogenetic Diversity | Collembola | Land use | 4 | 2.798 | 0.699 | 1.515 | 0.089 | 0.209 |
|  |  | Residuals | 62 | 28.632 | 0.462 |  | 0.911 |  |
|  | Coleoptera | Land use | 4 | 28.484 | 7.121 | 3.137 | 0.166 | 0.020 |
|  |  | Residuals | 63 | 143.012 | 2.270 |  | 0.834 |  |
|  | Diptera | Land use | 4 | 45.320 | 11.330 | 5.434 | 0.257 | 0.001 |
|  |  | Residuals | 63 | 131.343 | 2.085 |  | 0.743 |  |
|  | Hymenoptera | Land use | 4 | 28.103 | 7.026 | 4.138 | 0.213 | 0.005 |
|  |  | Residuals | 61 | 103.575 | 1.698 |  | 0.787 |  |
|  | Lepidoptera | Land use | 4 | 52.453 | 13.113 | 6.410 | 0.289 | < 0.001 |
|  |  | Residuals | 63 | 128.873 | 2.046 |  | 0.711 |  |
|  | Hemiptera | Land use | 4 | 14.560 | 3.640 | 2.571 | 0.144 | 0.047 |
|  |  | Residuals | 61 | 86.359 | 1.416 |  | 0.856 |  |
|  | other insects | Land use | 4 | 21.162 | 5.291 | 3.844 | 0.201 | 0.008 |
|  |  | Residuals | 61 | 83.945 | 1.376 |  | 0.799 |  |
|  | non-mites | Land use | 4 | 16.812 | 4.203 | 2.135 | 0.121 | 0.087 |
|  |  | Residuals | 62 | 122.060 | 1.969 |  | 0.879 |  |
|  | mites | Land use | 4 | 13.998 | 3.500 | 1.268 | 0.074 | 0.292 |
|  |  | Residuals | 63 | 173.902 | 2.760 |  | 0.926 |  |
|  | Malacostraca | Land use | 4 | 1.875 | 0.469 | 0.739 | 0.087 | 0.573 |
|  |  | Residuals | 31 | 19.662 | 0.634 |  | 0.913 |  |
|  | myriapods | Land use | 4 | 0.766 | 0.191 | 0.374 | 0.049 | 0.825 |
|  |  | Residuals | 29 | 14.850 | 0.512 |  | 0.951 |  |
|  | Annelida | Land use | 4 | 11.407 | 2.852 | 3.551 | 0.189 | 0.011 |
|  |  | Residuals | 61 | 48.988 | 0.803 |  | 0.811 |  |
|  | Mollusca | Land use | 4 | 27.760 | 6.940 | 2.741 | 0.152 | 0.037 |
|  |  | Residuals | 61 | 154.469 | 2.532 |  | 0.848 |  |
|  | Nematoda | Land use | 4 | 51.134 | 12.783 | 3.214 | 0.167 | 0.018 |
|  |  | Residuals | 64 | 254.528 | 3.977 |  | 0.833 |  |
|  | Platyhelminthes | Land use | 4 | 4.965 | 1.241 | 2.064 | 0.152 | 0.101 |
|  |  | Residuals | 46 | 27.655 | 0.601 |  | 0.848 |  |
|  | Rotifera | Land use | 4 | 7.680 | 1.920 | 0.912 | 0.055 | 0.462 |
|  |  | Residuals | 63 | 132.574 | 2.104 |  | 0.945 |  |
|  | Tardigrada | Land use | 4 | 0.448 | 0.112 | 1.006 | 0.130 | 0.422 |
|  |  | Residuals | 27 | 3.006 | 0.111 |  | 0.870 |  |
| Phylogenetic Rarity | Collembola | Land use | 4 | 0.263 | 0.066 | 1.604 | 0.094 | 0.185 |
|  |  | Residuals | 62 | 2.542 | 0.041 |  | 0.906 |  |
|  | Coleoptera | Land use | 4 | 8.623 | 2.156 | 6.726 | 0.299 | < 0.001 |
|  |  | Residuals | 63 | 20.192 | 0.321 |  | 0.701 |  |
|  | Diptera | Land use | 4 | 11.176 | 2.794 | 11.067 | 0.413 | < 0.001 |
|  |  | Residuals | 63 | 15.905 | 0.252 |  | 0.587 |  |
|  | Hymenoptera | Land use | 4 | 3.729 | 0.932 | 4.937 | 0.245 | 0.002 |
|  |  | Residuals | 61 | 11.520 | 0.189 |  | 0.755 |  |
|  | Lepidoptera | Land use | 4 | 7.029 | 1.757 | 8.995 | 0.364 | < 0.001 |
|  |  | Residuals | 63 | 12.307 | 0.195 |  | 0.636 |  |
|  | Hemiptera | Land use | 4 | 1.089 | 0.272 | 2.484 | 0.140 | 0.053 |
|  |  | Residuals | 61 | 6.685 | 0.110 |  | 0.860 |  |
|  | other insects | Land use | 4 | 2.844 | 0.711 | 3.912 | 0.204 | 0.007 |
|  |  | Residuals | 61 | 11.088 | 0.182 |  | 0.796 |  |
|  | non-mites | Land use | 4 | 6.253 | 1.563 | 6.824 | 0.306 | < 0.001 |
|  |  | Residuals | 62 | 14.203 | 0.229 |  | 0.694 |  |
|  | mites | Land use | 4 | 2.379 | 0.595 | 2.740 | 0.148 | 0.036 |
|  |  | Residuals | 63 | 13.676 | 0.217 |  | 0.852 |  |
|  | Malacostraca | Land use | 4 | 0.204 | 0.051 | 0.772 | 0.091 | 0.552 |
|  |  | Residuals | 31 | 2.052 | 0.066 |  | 0.909 |  |
|  | myriapods | Land use | 4 | 0.276 | 0.069 | 1.558 | 0.177 | 0.212 |
|  |  | Residuals | 29 | 1.283 | 0.044 |  | 0.823 |  |
|  | Annelida | Land use | 4 | 2.097 | 0.524 | 6.507 | 0.299 | < 0.001 |
|  |  | Residuals | 61 | 4.915 | 0.081 |  | 0.701 |  |
|  | Mollusca | Land use | 4 | 3.365 | 0.841 | 2.186 | 0.125 | 0.081 |
|  |  | Residuals | 61 | 23.477 | 0.385 |  | 0.875 |  |
|  | Nematoda | Land use | 4 | 3.459 | 0.865 | 2.847 | 0.151 | 0.031 |
|  |  | Residuals | 64 | 19.443 | 0.304 |  | 0.849 |  |
|  | Platyhelminthes | Land use | 4 | 0.219 | 0.055 | 0.951 | 0.076 | 0.443 |
|  |  | Residuals | 46 | 2.654 | 0.058 |  | 0.924 |  |
|  | Rotifera | Land use | 4 | 0.864 | 0.216 | 0.898 | 0.054 | 0.470 |
|  |  | Residuals | 63 | 15.157 | 0.241 |  | 0.946 |  |
|  | Tardigrada | Land use | 4 | 0.157 | 0.039 | 1.432 | 0.175 | 0.250 |
|  |  | Residuals | 27 | 0.739 | 0.027 |  | 0.825 |  |
| Mean Pairwise Distance | Collembola | Land use | 4 | 0.038 | 0.009 | 0.394 | 0.025 | 0.812 |
|  |  | Residuals | 62 | 1.491 | 0.024 |  | 0.975 |  |
|  | Coleoptera | Land use | 4 | 0.036 | 0.009 | 0.434 | 0.027 | 0.784 |
|  |  | Residuals | 63 | 1.304 | 0.021 |  | 0.973 |  |
|  | Diptera | Land use | 4 | 0.078 | 0.020 | 0.535 | 0.033 | 0.710 |
|  |  | Residuals | 63 | 2.301 | 0.037 |  | 0.967 |  |
|  | Hymenoptera | Land use | 4 | 1.240 | 0.310 | 6.240 | 0.290 | < 0.001 |
|  |  | Residuals | 61 | 3.031 | 0.050 |  | 0.710 |  |
|  | Lepidoptera | Land use | 4 | 0.109 | 0.027 | 0.777 | 0.047 | 0.545 |
|  |  | Residuals | 63 | 2.218 | 0.035 |  | 0.953 |  |
|  | Hemiptera | Land use | 4 | 0.324 | 0.081 | 0.858 | 0.053 | 0.494 |
|  |  | Residuals | 61 | 5.757 | 0.094 |  | 0.947 |  |
|  | other insects | Land use | 4 | 0.275 | 0.069 | 1.118 | 0.068 | 0.356 |
|  |  | Residuals | 61 | 3.750 | 0.061 |  | 0.932 |  |
|  | non-mites | Land use | 4 | 0.257 | 0.064 | 0.828 | 0.051 | 0.512 |
|  |  | Residuals | 62 | 4.801 | 0.077 |  | 0.949 |  |
|  | mites | Land use | 4 | 0.492 | 0.123 | 3.527 | 0.183 | 0.012 |
|  |  | Residuals | 63 | 2.197 | 0.035 |  | 0.817 |  |
|  | Malacostraca | Land use | 4 | 1.644 | 0.411 | 1.157 | 0.130 | 0.349 |
|  |  | Residuals | 31 | 11.013 | 0.355 |  | 0.870 |  |
|  | myriapods | Land use | 4 | 0.642 | 0.161 | 0.684 | 0.086 | 0.609 |
|  |  | Residuals | 29 | 6.813 | 0.235 |  | 0.914 |  |
|  | Annelida | Land use | 4 | 0.464 | 0.116 | 1.997 | 0.116 | 0.106 |
|  |  | Residuals | 61 | 3.546 | 0.058 |  | 0.884 |  |
|  | Mollusca | Land use | 4 | 0.335 | 0.084 | 0.921 | 0.057 | 0.458 |
|  |  | Residuals | 61 | 5.544 | 0.091 |  | 0.943 |  |
|  | Nematoda | Land use | 4 | 0.033 | 0.008 | 1.023 | 0.060 | 0.402 |
|  |  | Residuals | 64 | 0.519 | 0.008 |  | 0.940 |  |
|  | Platyhelminthes | Land use | 4 | 1.565 | 0.391 | 1.105 | 0.088 | 0.365 |
|  |  | Residuals | 46 | 16.288 | 0.354 |  | 0.912 |  |
|  | Rotifera | Land use | 4 | 0.011 | 0.003 | 5.848 | 0.271 | < 0.001 |
|  |  | Residuals | 63 | 0.029 | 0.000 |  | 0.729 |  |
|  | Tardigrada | Land use | 4 | 0.469 | 0.117 | 1.229 | 0.154 | 0.322 |
|  |  | Residuals | 27 | 2.575 | 0.095 |  | 0.846 |  |
