# Supplementary material for: Rarity is a more reliable indicator of land-use impacts on soil invertebrate communities than other diversity metrics
Source: eLife. 2020 May 19;9:e52787. doi: 10.7554/eLife.52787 (PMC7237214; doi:10.7554/eLife.52787)
Supplement: Figure 4—source data 1. [file elife-52787-fig4-data1.docx]

Figure 4-source data 1

A. Results of ANOVA tests for overall soil invertebrate phylogenetic biodiversity SES differences between land-use categories. Tests resulting in *P*-values ≤ 0.05 are highlighted.

| **Metric** | **Term** | **Df** | **Sum Sq.** | **Mean Sq.** | ***F* stat.** | **R^2^** | ***P*** |
| --- | --- | --- | --- | --- | --- | --- | --- |
| Phylogenetic Diversity SES | Land use | 4 | 59.464 | 14.866 | 3.503 | 0.180 | 0.012 |
|  | Residuals | 64 | 271.628 | 4.244 |  | 0.820 |  |
| Phylogenetic Rarity SES | Land use | 4 | 689.222 | 172.306 | 17.986 | 0.529 | < 0.001 |
|  | Residuals | 64 | 613.133 | 9.580 |  | 0.471 |  |
| Mean Pairwise Distance SES | Land use | 4 | 23.966 | 5.992 | 1.782 | 0.100 | 0.143 |
|  | Residuals | 64 | 215.197 | 3.362 |  | 0.900 |  |

B. Results of ANOVA tests for phylogenetic biodiversity SES differences between land-use categories for different invertebrate groups. ‘Non-mites’ consists of Araneae, Opiliones, and Pseudoscorpiones. ‘Other insects’ consists of all insect orders other than Coleoptera, Diptera, Hemiptera, Hymenoptera, and Lepidoptera. Tests resulting in *P*-values ≤ 0.05 are highlighted.

| **Metric** | **Taxon** | **Term** | **Df** | **Sum Sq.** | **Mean Sq.** | ***F* stat.** | **R^2^** | ***P*** |
| --- | --- | --- | --- | --- | --- | --- | --- | --- |
| Phylogenetic  Diversity SES | Collembola | Land use | 4 | 7.411 | 1.853 | 0.571 | 0.037 | 0.685 |
|  |  | Residuals | 59 | 191.589 | 3.247 |  | 0.963 |  |
|  | Coleoptera | Land use | 4 | 4.483 | 1.121 | 0.629 | 0.038 | 0.644 |
|  |  | Residuals | 63 | 112.274 | 1.782 |  | 0.962 |  |
|  | Diptera | Land use | 4 | 17.597 | 4.399 | 1.439 | 0.086 | 0.232 |
|  |  | Residuals | 61 | 186.516 | 3.058 |  | 0.914 |  |
|  | Hymenoptera | Land use | 4 | 27.202 | 6.800 | 4.455 | 0.235 | 0.003 |
|  |  | Residuals | 58 | 88.531 | 1.526 |  | 0.765 |  |
|  | Lepidoptera | Land use | 4 | 21.080 | 5.270 | 5.893 | 0.279 | < 0.001 |
|  |  | Residuals | 61 | 54.551 | 0.894 |  | 0.721 |  |
|  | Hemiptera | Land use | 4 | 11.591 | 2.898 | 1.272 | 0.083 | 0.292 |
|  |  | Residuals | 56 | 127.532 | 2.277 |  | 0.917 |  |
|  | other insects | Land use | 4 | 5.774 | 1.444 | 1.121 | 0.072 | 0.355 |
|  |  | Residuals | 58 | 74.670 | 1.287 |  | 0.928 |  |
|  | non-mites | Land use | 4 | 8.762 | 2.191 | 1.090 | 0.069 | 0.370 |
|  |  | Residuals | 59 | 118.590 | 2.010 |  | 0.931 |  |
|  | mites | Land use | 4 | 70.553 | 17.638 | 5.600 | 0.265 | < 0.001 |
|  |  | Residuals | 62 | 195.266 | 3.149 |  | 0.735 |  |
|  | Malacostraca | Land use | 4 | 21.493 | 5.373 | 4.418 | 0.576 | 0.018 |
|  |  | Residuals | 13 | 15.812 | 1.216 |  | 0.424 |  |
|  | myriapods | Land use | 4 | 5.757 | 1.439 | 0.526 | 0.161 | 0.719 |
|  |  | Residuals | 11 | 30.081 | 2.735 |  | 0.839 |  |
|  | Annelida | Land use | 4 | 18.748 | 4.687 | 1.295 | 0.082 | 0.283 |
|  |  | Residuals | 58 | 209.877 | 3.619 |  | 0.918 |  |
|  | Mollusca | Land use | 4 | 16.339 | 4.085 | 2.094 | 0.128 | 0.093 |
|  |  | Residuals | 57 | 111.169 | 1.950 |  | 0.872 |  |
|  | Nematoda | Land use | 4 | 17.123 | 4.281 | 2.763 | 0.147 | 0.035 |
|  |  | Residuals | 64 | 99.165 | 1.549 |  | 0.853 |  |
|  | Platyhelminthes | Land use | 4 | 10.414 | 2.603 | 2.696 | 0.375 | 0.064 |
|  |  | Residuals | 18 | 17.380 | 0.966 |  | 0.625 |  |
|  | Rotifera | Land use | 4 | 55.334 | 13.833 | 7.385 | 0.319 | < 0.001 |
|  |  | Residuals | 63 | 118.008 | 1.873 |  | 0.681 |  |
|  | Tardigrada | Land use | 4 | 110.403 | 27.601 | 1.130 | 0.475 | 0.437 |
|  |  | Residuals | 5 | 122.077 | 24.415 |  | 0.525 |  |
| Phylogenetic  Rarity SES | Collembola | Land use | 4 | 101.814 | 25.453 | 7.796 | 0.335 | < 0.001 |
|  |  | Residuals | 62 | 202.427 | 3.265 |  | 0.665 |  |
|  | Coleoptera | Land use | 4 | 83.632 | 20.908 | 5.179 | 0.247 | 0.001 |
|  |  | Residuals | 63 | 254.330 | 4.037 |  | 0.753 |  |
|  | Diptera | Land use | 4 | 124.326 | 31.082 | 8.459 | 0.349 | < 0.001 |
|  |  | Residuals | 63 | 231.498 | 3.675 |  | 0.651 |  |
|  | Hymenoptera | Land use | 4 | 19.674 | 4.919 | 1.067 | 0.065 | 0.381 |
|  |  | Residuals | 61 | 281.118 | 4.608 |  | 0.935 |  |
|  | Lepidoptera | Land use | 4 | 60.716 | 15.179 | 7.827 | 0.332 | < 0.001 |
|  |  | Residuals | 63 | 122.180 | 1.939 |  | 0.668 |  |
|  | Hemiptera | Land use | 4 | 23.178 | 5.795 | 2.473 | 0.140 | 0.054 |
|  |  | Residuals | 61 | 142.937 | 2.343 |  | 0.860 |  |
|  | other insects | Land use | 4 | 37.525 | 9.381 | 3.100 | 0.169 | 0.022 |
|  |  | Residuals | 61 | 184.606 | 3.026 |  | 0.831 |  |
|  | non-mites | Land use | 4 | 93.916 | 23.479 | 7.560 | 0.328 | < 0.001 |
|  |  | Residuals | 62 | 192.541 | 3.106 |  | 0.672 |  |
|  | mites | Land use | 4 | 106.643 | 26.661 | 8.914 | 0.361 | < 0.001 |
|  |  | Residuals | 63 | 188.430 | 2.991 |  | 0.639 |  |
|  | Malacostraca | Land use | 4 | 4.597 | 1.149 | 0.553 | 0.067 | 0.698 |
|  |  | Residuals | 31 | 64.388 | 2.077 |  | 0.933 |  |
|  | myriapods | Land use | 4 | 81.877 | 20.469 | 1.793 | 0.198 | 0.157 |
|  |  | Residuals | 29 | 331.162 | 11.419 |  | 0.802 |  |
|  | Annelida | Land use | 4 | 78.251 | 19.563 | 5.035 | 0.248 | 0.001 |
|  |  | Residuals | 61 | 237.001 | 3.885 |  | 0.752 |  |
|  | Mollusca | Land use | 4 | 50.094 | 12.524 | 6.659 | 0.304 | < 0.001 |
|  |  | Residuals | 61 | 114.719 | 1.881 |  | 0.696 |  |
|  | Nematoda | Land use | 4 | 54.693 | 13.673 | 3.768 | 0.191 | 0.008 |
|  |  | Residuals | 64 | 232.266 | 3.629 |  | 0.809 |  |
|  | Platyhelminthes | Land use | 4 | 3.600 | 0.900 | 0.569 | 0.047 | 0.687 |
|  |  | Residuals | 46 | 72.810 | 1.583 |  | 0.953 |  |
|  | Rotifera | Land use | 4 | 67.258 | 16.814 | 5.318 | 0.252 | 0.001 |
|  |  | Residuals | 63 | 199.184 | 3.162 |  | 0.748 |  |
|  | Tardigrada | Land use | 4 | 28.871 | 7.218 | 1.226 | 0.154 | 0.323 |
|  |  | Residuals | 27 | 158.927 | 5.886 |  | 0.846 |  |
| Mean Pairwise  Distance SES | Collembola | Land use | 4 | 5.762 | 1.440 | 0.461 | 0.030 | 0.764 |
|  |  | Residuals | 59 | 184.448 | 3.126 |  | 0.970 |  |
|  | Coleoptera | Land use | 4 | 9.199 | 2.300 | 1.089 | 0.065 | 0.370 |
|  |  | Residuals | 63 | 133.091 | 2.113 |  | 0.935 |  |
|  | Diptera | Land use | 4 | 13.217 | 3.304 | 1.213 | 0.074 | 0.315 |
|  |  | Residuals | 61 | 166.210 | 2.725 |  | 0.926 |  |
|  | Hymenoptera | Land use | 4 | 6.141 | 1.535 | 2.242 | 0.134 | 0.075 |
|  |  | Residuals | 58 | 39.711 | 0.685 |  | 0.866 |  |
|  | Lepidoptera | Land use | 4 | 46.548 | 11.637 | 11.275 | 0.425 | < 0.001 |
|  |  | Residuals | 61 | 62.958 | 1.032 |  | 0.575 |  |
|  | Hemiptera | Land use | 4 | 9.150 | 2.287 | 0.932 | 0.062 | 0.452 |
|  |  | Residuals | 56 | 137.392 | 2.453 |  | 0.938 |  |
|  | other insects | Land use | 4 | 8.307 | 2.077 | 1.428 | 0.090 | 0.236 |
|  |  | Residuals | 58 | 84.368 | 1.455 |  | 0.910 |  |
|  | non-mites | Land use | 4 | 4.041 | 1.010 | 0.520 | 0.034 | 0.721 |
|  |  | Residuals | 59 | 114.630 | 1.943 |  | 0.966 |  |
|  | mites | Land use | 4 | 49.247 | 12.312 | 4.400 | 0.221 | 0.003 |
|  |  | Residuals | 62 | 173.497 | 2.798 |  | 0.779 |  |
|  | Malacostraca | Land use | 4 | 22.774 | 5.694 | 4.578 | 0.585 | 0.016 |
|  |  | Residuals | 13 | 16.166 | 1.244 |  | 0.415 |  |
|  | myriapods | Land use | 4 | 10.698 | 2.675 | 0.724 | 0.208 | 0.594 |
|  |  | Residuals | 11 | 40.650 | 3.695 |  | 0.792 |  |
|  | Annelida | Land use | 4 | 34.103 | 8.526 | 2.116 | 0.127 | 0.090 |
|  |  | Residuals | 58 | 233.741 | 4.030 |  | 0.873 |  |
|  | Mollusca | Land use | 4 | 16.404 | 4.101 | 2.472 | 0.148 | 0.055 |
|  |  | Residuals | 57 | 94.567 | 1.659 |  | 0.852 |  |
|  | Nematoda | Land use | 4 | 9.925 | 2.481 | 1.585 | 0.090 | 0.189 |
|  |  | Residuals | 64 | 100.185 | 1.565 |  | 0.910 |  |
|  | Platyhelminthes | Land use | 4 | 9.591 | 2.398 | 2.572 | 0.364 | 0.073 |
|  |  | Residuals | 18 | 16.779 | 0.932 |  | 0.636 |  |
|  | Rotifera | Land use | 4 | 64.473 | 16.118 | 8.676 | 0.355 | < 0.001 |
|  |  | Residuals | 63 | 117.034 | 1.858 |  | 0.645 |  |
|  | Tardigrada | Land use | 4 | 75.130 | 18.782 | 1.148 | 0.479 | 0.431 |
|  |  | Residuals | 5 | 81.792 | 16.358 |  | 0.521 |  |
