# Supplementary material for: Rarity is a more reliable indicator of land-use impacts on soil invertebrate communities than other diversity metrics
Source: eLife. 2020 May 19;9:e52787. doi: 10.7554/eLife.52787 (PMC7237214; doi:10.7554/eLife.52787)
Supplement: Figure 6—source data 1. [file elife-52787-fig6-data1.docx]

Figure 6-source data 1

Defining attributes of land-use categories.

| **Land-use category** | **Attributes** |
| --- | --- |
| Natural forest | Tall forest dominated by indigenous conifer, broadleaved or beech species. |
| Planted forest | Planted or naturalised forest predominantly of radiata pine but including other pine species, Douglas fir, cypress, larch, acacia and eucalypts. Production forestry is the main land use in this class with minor areas devoted to mass-movement erosion-control and other areas of naturalised (wildling) establishment. |
| Low-producing grassland | Exotic sward grassland and indigenous short tussock grassland of poor pastoral quality reflecting lower soil fertility and extensive grazing management or non-agricultural use. Browntop, sweet vernal, Danthonia, fescue and Yorkshire fog dominate, with indigenous short tussocks (hard tussock, blue tussock and silver tussock) common in the eastern South Island and locally elsewhere. |
| High-producing grassland | Exotic sward grassland of good pastoral quality and vigour reflecting relatively high soil fertility and intensive grazing management. Clover species, ryegrass and cocksfoot dominate with lucerne and plantain locally important, but also including lower-producing grasses exhibiting vigour in areas of good soil moisture and fertility. |
| Perennial cropland | Land managed for production of grapes, pip, citrus and stone fruit, nuts, olives, berries, kiwifruit, and other perennial crops. Cultivation for crop renewal is infrequent and irregular but is sometimes practiced for weed control. |
