# Supplementary material for: Rarity is a more reliable indicator of land-use impacts on soil invertebrate communities than other diversity metrics
Source: eLife. 2020 May 19;9:e52787. doi: 10.7554/eLife.52787 (PMC7237214; doi:10.7554/eLife.52787)
Supplement: Transparent reporting form [file elife-52787-transrepform.docx]

***eLife’s* transparent reporting form**

We encourage authors to provide detailed information *within their submission* to facilitate the interpretation and replication of experiments. Authors can upload supporting documentation to indicate the use of appropriate reporting guidelines for health-related research (see [EQUATOR Network](http://www.equator-network.org/%20)), life science research (see the [BioSharing Information Resource](https://biosharing.org/)), or the [ARRIVE guidelines](http://www.plosbiology.org/article/info:doi/10.1371/journal.pbio.1000412) for reporting work involving animal research. Where applicable, authors should refer to any relevant reporting standards documents in this form.

If you have any questions, please consult our Journal Policies and/or contact us: [editorial@elifesciences.org](mailto:editorial@elifesciences.org).

**Sample-size estimation**

- You should state whether an appropriate sample size was computed when the study was being designed
- You should state the statistical method of sample size computation and any required assumptions
- If no explicit power analysis was used, you should describe how you decided what sample (replicate) size (number) to use

Please outline where this information can be found within the submission (e.g., sections or figure legends), or explain why this information doesn’t apply to your submission:

Sample size was based on rules of thumb, as no comparable data on which to base a power analysis was available at the time that this experiment was designed. We sampled fifteen sites per land use (75 sites in total), expecting that further gains in statistical power due to additional samples would be small relative to the cost of doing so.

**Replicates**

- You should report how often each experiment was performed
- You should include a definition of biological versus technical replication
- The data obtained should be provided and sufficient information should be provided to indicate the number of independent biological and/or technical replicates
- If you encountered any outliers, you should describe how these were handled
- Criteria for exclusion/inclusion of data should be clearly stated
- High-throughput sequence data should be uploaded before submission, with a private link for reviewers provided (these are available from both GEO and ArrayExpress)

Please outline where this information can be found within the submission (e.g., sections or figure legends), or explain why this information doesn’t apply to your submission:

This information is provided throughout the methods section. Each sample plot was considered an independent biological replicate, and sampled once.

Outliers were not excluded, except for non-metric MDS ordinations, from which any unusually low abundance samples (those with abundance < 5 % of the mean abundance per sample) were excluded, and a single specific sample excluded from one particular MDS ordination, because its inclusion resulted in an uninterpretable figure.

Sequencing data has been made available at: https://datastore.landcareresearch.co.nz/dataset/soil-invertebrate-biodiversity-vs-landuse

**Statistical reporting**

- Statistical analysis methods should be described and justified
- Raw data should be presented in figures whenever informative to do so (typically when N per group is less than 10)
- For each experiment, you should identify the statistical tests used, exact values of N, definitions of center, methods of multiple test correction, and dispersion and precision measures (e.g., mean, median, SD, SEM, confidence intervals; and, for the major substantive results, a measure of effect size (e.g., Pearson's r, Cohen's d)
- Report exact p-values wherever possible alongside the summary statistics and 95% confidence intervals. These should be reported for all key questions and not only when the p-value is less than 0.05.

Please outline where this information can be found within the submission (e.g., sections or figure legends), or explain why this information doesn’t apply to your submission:

The statistical analysis methods used are described in the methods section.

Brief summaries of statistical test results including p-values are provided in the results section, with complete details in the source data tables and the appendix. Means and standard errors are provided in figures 3 and 4.

We also provide our analysis code, enabling others to review and reproduce our statistical analyses.

(For large datasets, or papers with a very large number of statistical tests, you may upload a single table file with tests, Ns, etc., with reference to sections in the manuscript.)

**Group allocation**

- Indicate how samples were allocated into experimental groups (in the case of clinical studies, please specify allocation to treatment method); if randomization was used, please also state if restricted randomization was applied
- Indicate if masking was used during group allocation, data collection and/or data analysis

Please outline where this information can be found within the submission (e.g., sections or figure legends), or explain why this information doesn’t apply to your submission:

This doesn’t apply to our submission. Our study does not involve any experimental manipulations or groups.

**Additional data files (“source data”)**

- We encourage you to upload relevant additional data files, such as numerical data that are represented as a graph in a figure, or as a summary table
- Where provided, these should be in the most useful format, and they can be uploaded as “Source data” files linked to a main figure or table
- Include model definition files including the full list of parameters used
- Include code used for data analysis (e.g., R, MatLab)
- Avoid stating that data files are “available upon request”

Please indicate the figures or tables for which source data files have been provided:

The raw sequence data, processed data (a table of OTU abundances; taxonomic identifications; a phylogenetic tree; and sample metadata), and the bioinformatic processing and data analysis code (Python and R) used to generate all our results are available from:

https://datastore.landcareresearch.co.nz/dataset/soil-invertebrate-biodiversity-vs-landuse

This enables others to reproduce all of our results.
